# Supplementary material for: Genetic diversity and drug resistance pattern of Mycobacterium tuberculosis strains isolated from pulmonary tuberculosis patients in the Benishangul Gumuz region and its surroundings, Northwest Ethiopia
Source: PLoS One. 2020 Apr 8;15(4):e0231320. doi: 10.1371/journal.pone.0231320 (PMC7141659; doi:10.1371/journal.pone.0231320)
Supplement: S1 Fig — (PDF) [file pone.0231320.s001.pdf]

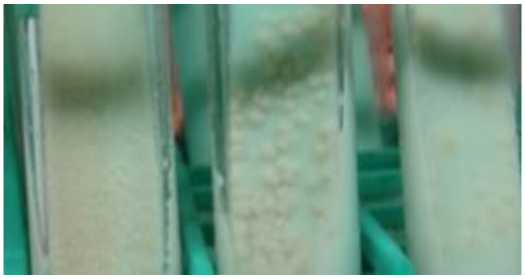

S1 Fig. Colony characteristic used for identification of the growth of *Mycobacterium tuberculosis* on egg based Löwenstein-Jensen *(LJ)* medium. Sputum samples were collected from AFB smear positive TB patients in Benishangul Gumuz region and its surroundings in North West Ethiopia.
