# Supplementary material for: Genetic diversity and drug resistance pattern of Mycobacterium tuberculosis strains isolated from pulmonary tuberculosis patients in the Benishangul Gumuz region and its surroundings, Northwest Ethiopia
Source: PLoS One. 2020 Apr 8;15(4):e0231320. doi: 10.1371/journal.pone.0231320 (PMC7141659; doi:10.1371/journal.pone.0231320)
Supplement: S2 Fig — (PDF) [file pone.0231320.s002.pdf]

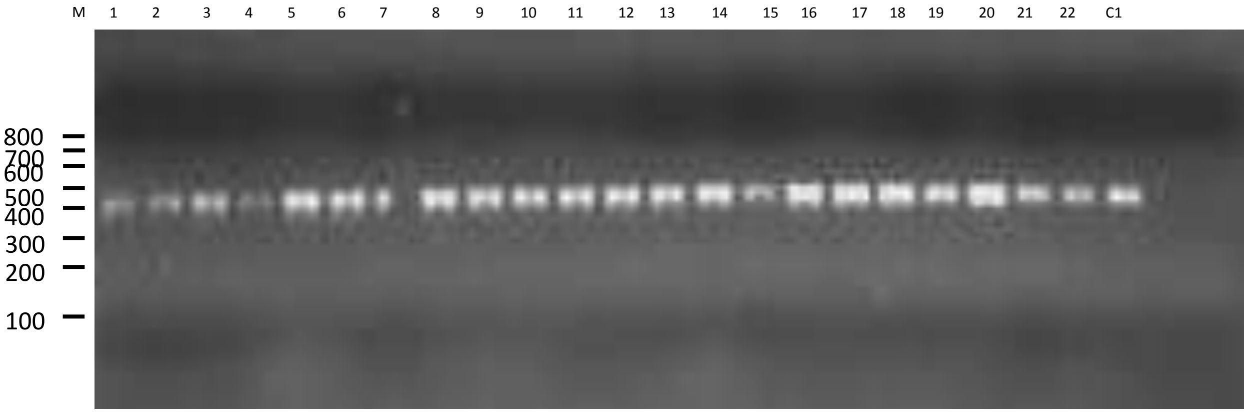

S2 Fig. PCR for RD9 Deletion typing (M= Marker with 100bp size, Lanes 1-22=Clinical isolates, Lane C1= H37Rv _ATCC 27294 (Positive control). The clinical samples were from AFB smear positive TB suspects in Benishangul Gumuz region and its surroundings in North West Ethiopia.
