# Supplementary material for: Genetic diversity and drug resistance pattern of Mycobacterium tuberculosis strains isolated from pulmonary tuberculosis patients in the Benishangul Gumuz region and its surroundings, Northwest Ethiopia
Source: PLoS One. 2020 Apr 8;15(4):e0231320. doi: 10.1371/journal.pone.0231320 (PMC7141659; doi:10.1371/journal.pone.0231320)
Supplement: S1 Table — A and B. Frequency of M. tuberculosis family and Lineages. (PDF) [file pone.0231320.s003.pdf]

Table A:

Lineage	Family	N(%)	N(%)
EA	T1	12 (16.0)	
	T3	9 (12.0)	
	T3-ETH	4 (5.3)	
	H3	4 (5.3)	
	T2	2 (2.7)	
	H1	2 (2.7)	
	T	1 (1.3)	
	X1	1 (1.3)	
	LAM5	1 (1.3)	
	Manu2	1 (1.3)	
	ND	15(20)	
	Unknown	2(2.7)	54(72)
EAI	CAS1-Delhi	11 (14.7)	
	CAS1-Kili	1 (1.3)	
	ND	7(9.33)	19(25,3)
EAS	ND	1(1.3)	1(1.3)
IO	ND	1(1.3)	1(1.3)
**Total**		75 (100)	75 (100)

EA: Euro American **EAI**: East African Indian **EAS**: East Asian **IO**: Indo-Oceanic
ND : Not defined yet; **Unknown**: strains with SIT but not further classified

Table B:

Family	Sub-family	N(%)	Total N (%)
T	T	1(1,3)	
	T1	12(16,0)	
	T2	2(2,7)	
	T3	9(12)	
	T3-ETH	4(5,3)	28(37,3)
H			
	H1	2(2,7)	
	H3	4(5,3)	6(8,0)
CAS	CAS1-Delhi	11(14,7)	
	CAS1-Kili	1(1,3)	12(16,0)
Others			
	X1	1(1,3)	1(1,3)
	LAM5	1(1,3)	1(1,3)
	Manu2	1(1,3)	1(1,3)
	Undefined*	24(32,0)	24(32,0)
	Unknown**	2(2.7)	2(2.7)
**Total**			**75(100)**

* **Undefined:** Strains not defined yet ****Unknown**: Strains with defined SIT but not grouped into Family
