# Supplementary material for: Genetic diversity and drug resistance pattern of Mycobacterium tuberculosis strains isolated from pulmonary tuberculosis patients in the Benishangul Gumuz region and its surroundings, Northwest Ethiopia
Source: PLoS One. 2020 Apr 8;15(4):e0231320. doi: 10.1371/journal.pone.0231320 (PMC7141659; doi:10.1371/journal.pone.0231320)
Supplement: Attachment — Submitted filename: Main manuscript_With track changes.docx [file pone.0231320.s004.docx]

**Genetic Diversity and Drug Resistance Pattern of *Mycobacterium tuberculosis* Strains from Pulmonary Tuberculosis Patients in Benishangul Gumuz Region and Its Surroundings, Northwest Ethiopia**

Tekle Airgecho Lobie^1,2,3*^, Yimtubezinash Woldeamanuel^2^, Daniel Asrat^2^, Demissew Beyene^1,^ Magnar Bjørås^3, 4^, Abraham Aseffa^1^

^1^ Armauer Hansen Research Institute (AHRI), Addis Ababa, Ethiopia

^2^ Department of Microbiology, Immunology, and Parasitology (DMIP), Addis Ababa University, Addis Ababa, Ethiopia

^3^ Department of Microbiology, Oslo University Hospital and University of Oslo, Rikshospitalet, Oslo, Norway

^4^ Department of Clinical and Molecular Medicine, Norwegian University of Science and Technology, Trondheim, Norway

*Corresponding author: [teklemicro@gmail.com](mailto:teklemicro@gmail.com)

Abstract

**Introduction**

Tuberculosis remains a major global public health problem and the leading cause of death from a single bacterium, *Mycobacterium tuberculosis* complex. The emergence and spread of drug-resistant strains aggravates the problem especially to tuberculosis high burden countries, like Ethiopia. As a result of the supposedly high initial cost, there is limited or no information about drug resistance pattern and circulating strains in peripheral areas. Here we investigated drug susceptibility pattern and genetic diversity of mycobacterial strains circulating among pulmonary tuberculosis patients in Benishangul Gumuz region and its surroundings, northwest Ethiopia.

**Methods and material**

In a cross-sectional study, 107 consecutive sputum smear-positive pulmonary tuberculosis patients diagnosed from two hospitals and seven health centres between October 2013 and June 2014 were enrolled. Sputum samples were cultured at Armauer Hansen research institute (AHRI) tuberculosis laboratory and drug susceptibility testing done Isoniazid, Rifampicin, Ethambutol, and Streptomycin by indirect proportion method on Middlebrooks 7H10 medium. Isolates were characterized Polymerase chain reaction-based Region of Difference 9 (RD9) and spoligotyping.. Statistical analysis was performed using SPSS v.24.

**Results**

A total of 107 acid fast positive sputum samples were collected and up on culturing a positive culture yield of 81.3% (87/107) was achieved from samples that have been stored at the periphery and transported to the central laboratory. Using PCR based RD9 all the 87 isolates were confirmed as *Mycobacterium tuberculosis*. Resistance to one or more drug was observed in 16.1% (14/87).Isoniazid monoresistance occurred in 6.9% (6/87). Multidrug resistance was seen in two isolates (2.3%)one of which was resistant for all the four of drugs tested. Molecular typing revealed that the majority, 61.3% (46/75) of strains could be into ten spoligotype clusters containing 2-11 isolates each while the remaining 38.7%, (29/75) strains were unique. SIT289 and SIT53 constituted 24% and 19.6% respectively, while 29.3% (22/75) were ‘’New’’ to the database. The dominant families were T37%(28/75) CAS 16.0% (12/75) and H 8% (6/75) accounting to 51.3% (46/75) of all isolates.

**Conclusion and recommendations**

The current study indicated a moderate level of multidrug-resistant tuberculosis. However, the observed high monoresistance to Isoniazid is a potential threat for sudden increase of MDR-TB. Clustering spoligotype patterns suggest on-going active tuberculosis transmission of tuberculosis in the area. The results underscore the need for enhanced monitoring of drug resistance in this and other peripheral regions of the country using robust molecular tools with high discriminatory power.

**Introduction**

Despite advances in research and development, and availability of anti-tuberculosis drugs that cure most cases, tuberculosis (TB) remains a major global health problem, ranked as a leading cause of death from a single bacterial agent (1, 2).

World health organization (WHO)’s latest global report, there were 10 million incident cases of TB in 2017 with 1.3 million deaths among HIV-negative people, and an additional 300 000 deaths among HIV-positive, of which 90% were adults. In 2017, 558 000 new TB cases with resistance to rifampicin (RRTB) were reported, of which 82% or 460 000 had multidrug-resistance to TB (MDR-TB) (1).

Ethiopia had an estimated incidence of 223, prevalence of 212, and TB death of 32 per 100,000 with estimated MDR-TB rates of 1.6% and 12% among new cases and retreatment cases, respectively (3), (4).

(The recent national TB drug resistance survey estimated a rate of 2.3% MDR-TB among new cases which indicated a significant increase from 1.6% from the first-round report (5),(3). A major challenge is the low coverage of second level laboratory facilities as shown by the fact that only 1% of bacteriologically confirmed new TB cases and only 4.4% of retreatment cases had DST results (4).

Despite the slightly decreasing number of reported drug-susceptible TB cases (1), the increasing number of MDR-TB shows the need for better progress. Emergence and spread of drug-resistant TB is a result of interdependent factors emanating from patient (poor adherence, defaulting from treatment, and compromised immunity), health system (delayed or poor detection, poor treatment, lack or poor service when available) (6), and the bacteria’s natural selection pressure to enter into more severe untreatable and undetectable form within the host. Notably, *M. tuberculosis* survives under various stress conditions through long-term dormancy mainly modulated by its Toxin-antitoxin systems (7), (8)*.*

During the last few years, the introduction of several genotyping methods facilitated molecular epidemiologic studies on TB (9, 10) with large sets of databases and applications now available including for classification of clinical isolates into phylogenetic lineages at strain level (11, 12). These methods combined with drug susceptibility testing (DST) are becoming valuable tools to understand the distribution and transmission dynamics of drug-susceptible, MDR and XDR TB strains.

This study was designed to assess the drug susceptibility pattern and genetic diversity of *Mycobacterium tuberculosis* isolates from pulmonary TB patients in one of the peripheral regions of Ethiopia, Benishangul Gumuz, and its surroundings where infrastructure and health service coverage are limited.

**Materials and Methods**

**The Study settings**

This study was conducted in the Benishangul Gumuz region and its surroundings, about 700 KMs from Addis Ababa adjacent to Ethio-Sudan border in the northwest of the country. The total population of the region is estimated to be 976,000 in 2014 according to the projection based on the 2007 CSA census (13). About 30 % of the population lives below the national poverty level. The region is characterized as emerging and has limited infrastructure including health service facilities (14).

There are three administrative zones, namely Assosa, Metekel, and Kemashi. Metekel zone is the largest covering 26,272 Km^2^ which is half the area of the region.

The region has two hospitals (one additional hospital is being built in 2018) and 32 health centres. There were no TB culture and drug susceptibility testing laboratories in the region. Microscopy for detection of acid-fast bacilli is routinely available at both hospitals and in the four health centres included in the study. Additional three health centres were included from Awi zone of Amhara region which is geographically proximal to the study area (Fig 1).

***Figure 1 │ Map of the study area, Benishangul Gumuz Region and its surroundings.*** *Health institutions included in the current study are indicated by a house symbol H, and the hospitals encircled red. Dots represent towns in the region.*

**Study design**

A cross-sectional study was conducted from October 2013 - June 2014. The study participants were newly diagnosed sputum smear-positive pulmonary TB cases from facilities with TB clinics and AFB microscopy services. All pulmonary TB suspects sent to sputum examination were asked for informed consent and only patients with AFB positive smears were considered as eligible. Before data collection, a two days theoretical and practical training was given to data collectors mainly on the TB research ethics, documentation, safety, sample collection, storage, and patient handling.

**Data collection**

Sociodemographic and clinical data of participants were collected by a trained nurse working at TB clinics, and sputum sample was collected by a trained laboratory professional. HIV status was retrieved from the patient’s clinical record.

Participants were requested to give three sputum samples (Spot-Morning-Spot) as part of their routine diagnostic procedure and checked for AFB at the site of collection. Only AFB smear-positive sputum samples were pooled into 30 ml screw capped, leak-proof universal test tubes, and temporarily stored at -20 C until transported to AHRI TB laboratory as soon as possible, within not more than two weeks (15). Upon arrival to AHRI, sputum samples were processed immediately by using N-acetyl L- cysteine- sodium hydroxide (NALC-NaOH) method for sputum digestion and decontamination (16). Summary of laboratory workflow is depicted in Fig 2.

**Mycobacterial Culture**

Egg-based *Löwenstein*-*Jensen* (LJ) culture medium not more than one-month-old was used following WHO standard procedure (17). Briefly, after decontaminated and mixing with equal volume of NALC-NaOH, sputum samples were left standing for 15 minutes followed by centrifugation at 3000 rpm for 15 minutes. The supernatant was discarded and sediment was inoculated on two freshly prepared LJ medium slants containing 0.6% glycerol and one tube with 0.6% sodium pyruvate to facilitate the growth of *M.tuberculosis* and *M. bovis* respectively (17).

***Figure 2 │Flow chart indicating data collection and laboratory activities.***

Inoculated tubes were incubated at 37°C and followed-up weekly for eight weeks. Mycobacterial growth was confirmed by typical colony characteristic (S1 Fig) and AFB microscopy.

**Drug Susceptibility Testing**

Drug susceptibility testing was performed based on the indirect proportion method following standard procedures (18). Briefly, a 24-well agar plate was prepared with 2.5 ml of Middlebrook 7H10 medium supplemented with 10% OADC and 5% glycerol per well. Bacterial suspension was adjusted to McFarland standard 1 turbidity using 0.2µm filter sterilized MQ-H_2_O. Four drugs namely Isoniazid, Rifampicin, Streptomycin, and Ethambutol were used with minimum inhibitory concentration (MIC) breakpoints of 0.2μg/mL, 1μg/mL, 2μg/mL and 5μg/mL respectively. Two drug-free wells, one with 1: 100 dilution and another containing undiluted bacteria suspension, were included as controls.

A strain was considered as “Susceptible” if no growth or considerably less than 1% growth was observed on the well containing the MIC concentration of the corresponding drug compared to the control with 1 % inoculum; and “Resistant” when growth on the well with drug is greater than on the control with the 1% inoculum (19). Any contaminated or borderline results were repeated.

**Molecular typing using RD9 and Spoligotyping**

Mycobacterial species and *Mycobacterium tuberculosis* strain identification were carried out at AHRI TB molecular laboratory**.**

To obtain DNA for molecular typing, a loop full of a colony from LJ growth was resuspended in 50μl of 0.2µm filter sterilized 1% TE buffer in 2mL screw capped Nunc tubes. The suspension was heated at 80^o^C in the water bath for 45 minutes followed by sonication for an additional 15 minutes. The heat killed product was centrifuged to 13000 rpm for 10 minutes at 4^o^C and the supernatant was collected in a new tube.

*M. tuberculosis* was differentiated from other species of mycobacteria, based on PCR targeting the RD9 (20) and further typed into strains using spoligotyping based on polymorphism in the direct repeat (DR) locus of the mycobacterial chromosome (10),(20). A database with a large set of strains was used to generate octal codes, to assign SIT (Spoligotype International Type), their corresponding numbers, family and lineages of the isolates (12).

**Quality control**

*M. tuberculosis* H37Rv (ATCC 27294) and *M. bovis* (AF 61/2122/97) with known genotype and drug resistance pattern were run together during all laboratory assays including growth, detection, drug susceptibility testing, RD9 typing, and spoligotyping.

**Data analysis**

All socio-demographic and clinical data of the study participants’ and the laboratory test results were entered and analysed using SPSS version 24 (IBM, USA). Association between drug resistance, Strains type, and sociodemographic and clinical data were assessed. Statistical significance was considered at *P*-value less than 0.05.

**Results**

**Sociodemographic and clinical data**

From 107 smear-positive sputum samples collected, 81.3% (75/87) were culture positive and available for further analysis. Spoligotyping was not done for 12 isolates due to a technical problem and therefore only 75 were available for strain cluster analysis.

The mean age of participants was 29 ± 9.2 SD years and ranged from 15-65. Majority of the participants were between 18 and 47 years and account for 88.5% (77/87). Data on HIV status was available for 71.3% (62/87) among which16.1% (10/62) were positive. There were more female patients than male with 3:1 male to female ratio and the marital status of the participants was 55.2% married, 41.4% single, 2.3% widowed and 1.1% divorced (N=87) (Table 1).

**Table 1**│Sociodemographic and clinical characteristics of study participants from Benishangul Gumuz region and its surrounding, Northwest Ethiopia from October 2013-June 2014.

|  | **Characteristics** | **Variables** | **Frequency (N=87)**  **n (%)** |
| --- | --- | --- | --- |
| ***Socio-demographic characteristics*** | Gender | Male | **65 (74.7)** |
|  |  | Female | 22(25.3) |
|  | Residence | Urban | 22(25.3) |
|  |  | Rural | **65(74.7)** |
|  | Occupation | Farmer | **40(46)** |
|  |  | Merchant | 6(6.9) |
|  |  | Gov’t employee | 11(12.6) |
|  |  | Student | 8(9.2) |
|  |  | Daily labourer | 21(24.1) |
|  |  | Prisoner | 1(1.1) |
|  | Educational status | Illiterate | **34(39.1)** |
|  |  | Grade1-4 | 10(11.5) |
|  |  | Grade 5-8 | 18(20.7) |
|  |  | Grade 9-12 | 16(18.4) |
|  |  | Diploma and+ | 9(6.9) |
|  | Marital status | Married | **48(55.2)** |
|  |  | Single | 36(41.4) |
|  |  | Divorced | 1(1.1) |
|  |  | Widowed | 2(2.3) |
|  | Age groups | <18 | 5(5.7) |
|  |  | 18-27 | **38(43.7)** |
|  |  | 28-37 | 31(35.6) |
|  |  | 38-47 | 8(9.2) |
|  |  | 48-57 | 4(4.6) |
|  |  | >57 | 1(1.1) |
| ***Clinical characteristics*** | HIV Status | Positive | 10(11.5) |
|  |  | Negative | **52(59.8)** |
|  |  | Unknown | 25(28.7) |
|  | TB patient contact history | Yes | 20(23) |
|  |  | No | **51(58.6)** |
|  |  | Unknown | 16(18.4) |
|  | Previous anti-TB treatment | Yes | 12(13.8) |
|  |  | No | **73(83.9)** |
|  |  | Unknown | 2(2.3) |

**Anti-TB drug resistance pattern**

Among 87 *M.tuberculosis* isolates tested against Isoniazid, Rifampicin, Ethambutol and Streptomycin, as shown in Table 2, resistance to any of one drug was observed in 16.1% of the strains.

**Table 2** **|** Drug resistance pattern of first-line anti-TB drugs among pulmonary TB patients in the Benishangul Gumuz region and its surroundings, Northwest Ethiopia (N=87), 2013/14.

| Drug(s) tested | Susceptible  (N=87)  n (%) | Resistant  (N=87)  n (%) |
| --- | --- | --- |
| **Resistance to any one drug**  INH  RIF  ETB  STM | **73 (83.4)**  76(87.4)  85(97.7)  80(92.0)  85(97.7) | **14(16.1)**  11(12.6)  2(2.3)  7(8.0)  2(2.3) |
| **Resistance to only one drug**  INH  ETB  STM | **78(89.7)**  81(93.1)  85(97.7)  86(98.85) | **9(10.3)**  6(6.9)  2(2.3)  1(1.15) |
| **Resistance to only two drugs**  INH + ETB | 84(96.5) | 3(3.5) |
| **Resistance to three drugs only**  INH + ETB + RIF*** | 86(98.85) | 1(1.15) |
| **Resistance for all drugs tested**  INH + ETB+ RIF+ STM*** | 86(98.85) | 1(1.15) |
| **Polydrug resistance** | 82(94.25) | 5(5.75) |

**MDR-TB*: *M. tuberculosis* Strains resistant to at least Rifampicin and Isoniazid.

Multidrug-resistant TB was detected in 2.3% (2/87) with one of the strain resistant to all drugs tested. From the total of 87 isolates tested, proportion of monoresistance was 6.9%, 2.3%, and 1.15% for Isoniazid, Ethambutol, and Streptomycin respectively. There was no monoresistance to Rifampicin. Resistance to two drugs (Isoniazid and Ethambutol)was seen in 3.5%. Polydrug resistance (resistance to more than two drugs) was observed in 5.7% of isolates while triple drug resisnace was observed for 1.15% and involved Isoniazid, Ethambutol and Rifampicin.

**Molecular typing and genetic diversity**

PCR based RD typing revealed that all the 75 isolated investigated for spoligotyping were *M.tuberculosis* (S2 Fig). Using spoligotyping together with SITVIT2 (12) and SPOTCLUST (11) databases, binary and octal, SIT, family and Lineages were assigned (Fig 3). A total of 39 distinct spoligotype patterns with 61.3% (46/ 75) grouped in 10 similar spoligotyping patterns containing 2-11 isolates each, and 38.7% (29/75) represented as unique patterns. Lineage level classification revealed 72.0% (54/75), 25.3% (19/75), 1.3% (1/75) and 1.3% (1/75) isolates to be Euro American, EA (Lineage 4), East African Indian, EAL (Lineage 3), East Asian, EAS (Lineage 2) and Indo-Oceanic, IO (Lineage 1), respectively (S1 Table A). Three families, namely T 37.3% (28/75), CAS 16.0% (12/75),and H 8% (6/75) were predominant accounting for 61.3% (46/75) of the total mycobacterial isolates (S1 Table B). Among 61.3% (46/75) of the isolates showing similar spoligotype patterns, 71.7% (33/46) belonged to EA and 28.3% (13/46) to EAI, while 17% (8/46) were without shared international spoligotype (SIT) so far and thus labelled ‘'New''. Among the remaining (29/75) or 38.0% of the isolates that exhibited single uniwue spoligotyping pattern, 51.7% (15/29) have defined SIT while the rest 48.3% (14/29) were assigned as ‘’New’’.

**Figure 2: │Spoligotyping pattern of *M.tuberculosis* isolates from pulmonary TB patients from the Benishangul Gumuz region and its surroundings in 2013-14.**

Notably, SIT289 (11 isolates, CAS) and SIT53 (9 isolates, T1) were most frequent strains accounting for 14.7% (11/75)and 12% (9/75) respectively. None of the sociodemographic and clinical variables studied were found to be siginificantly associated with either drug resistance pattern or circulating strains isolated (Table 3).

**Table 3:** Bivariate logistic analysis of factors associated with resistance to any one of anti-TB drug

| **Variable** | | **Any drug resistance** | | **COR *(95%CI)*** | ***p-value*** |
| --- | --- | --- | --- | --- | --- |
|  |  | **Yes**  **N*(%*)*** | **No**  **N*(%*)*** |  |  |
| Gender | M | 8 *(12.3)* | 57 *(87.7)* | 1 |  |
|  | F | 6 *(28.3)* | 16 *(72.7)* | 0.37 *(0.11,1.24)* | 0.107 |
| Residence | Urban | 1 *(4.50)* | 21 *(95.5)* | 1 |  |
|  | Rural | 13 *(20.0)* | 52 *(80.0)* | 0.19 *(0.02 ,1.55)* | 0.121 |
| HIV status | Positive | 1 *(15.4)* | 9 *(84.6)* | 1 |  |
|  | Negative | 8 *(10.0)* | 44 *(90.0)* | 0.73 *(0.21,2.58)* | 0.614 |
|  | Unknown | 5 *(20.0)* | 20 *(80.0)* | 0.44 *(0.45 ,4.37)* | 0.487 |
| Previous treatment history | Yes | 5 *(31.3)* | 11 *(68.8)* | 1 |  |
|  | No | 9 *(12.7)* | 62 *(87.3)* | 3.13 *(0.88,11.12)* | 0.078 |
| Previous contact with TB patient | Yes | 6 *(22.2)* | 21 *(77.8)* | 1 |  |
|  | No | 8 *(13.3)* | 52 *(86.7)* | 1.86 *(0.58,6.0)* | 0.301 |
| Education status | Illiterate | 7 *(20.6)* | 27*(79.4)* | 1 |  |
|  | Literate | 7 *(13.2)* | 46 *(88.8)* | 1.70 *(0.54,5.38)* | 0.078 |
| Lineages | Lineage 1 (IO) | 0 (0.0) | 1(100.0) | NA | NA |
|  | Lineage 2 (EAS) | 0(0.0) | 1(100.0) | NA | NA |
|  | Lineage 3 (EAI) | 4(26.7) | 11(73.3) | NA | NA |
|  | Lineage 4 (EA) | 9(16.1) | 47(83.9) | 1.40 (0.043, 44.9) | 0.85 |
| Strains (SIT) | New | 5 (15.2) | 28 (84.8) | 1 |  |
|  | Previously defined | 8 (19.0) | 34 (91.0) | 0.76 (0.22,2.58) | 0.66 |

* % is calculated for row

**Discussion**

The main aim of this study was to understand the molecular epidemiology of *M.tuberculosis* strains circulating in a remote and hard-to-reach region in order to infer lessons for interventions in these and similar areas for which no such information exists. Benishangul Gumuz region and its surroundings are among the areas in Ethiopia where resources and infrastructures are severely limited. Sociodemographic and clinical information in relation to type of strains and their drug susceptibility patterns were analysed. Here, we showed results that either substantiated previous estimated extrapolated from other regions, added new evidence to support interventions or provided basiline data for further rigorous studies in the area.

All isolates identified were confirmed as *M.tuberculosis* by PCR based RD9 typing. This concurs with previous findings that have shown *M.tuberculosis* as responsible for more than 90 percent of pulmonary tuberculosis infections (22).

Spoligotyping revealed that 68% (51/75) of the strains with assigned SIT were in the latest database, SITIVIT2 (12). In comparison to the studies by other groups (23–25), the remaining 32% (24/75) accounts to a slightly higher proportion of ‘'New'' strains from the area indicating absence of prior studies in the area and in line with expected variation in strain distribution across different ecological niches (26).

The identified thirty-nine distinct spoligotype patterns constituting 61.3% (46/75) of isolates with similar spoligotyping pattern indicate a higher level of *M.tuberculosis* strain diversity in the area. The predominance of clusters within SIT289 (11 isolates) and SIT53 (9 isolates) with similar spoligotype patterns could strongly suggest an on-going active transmission of tuberculosis in the area. Remarkably, this is despite dispersed settlement of the population in the region.

SIT289 strain was previously reported as predominant in Gambella and Bahir Dar areas both of which shares long borders with the current study area (27-28). SIT53 is frequent in central north and south Ethiopia at Ambo*,* Bahir Dar, Dilla and Dessie (29). This study region has, despite its relative isolation, an increasingly mixed population including people originating from different parts of the country resettled in 1980s (30), which would contribute to the diversity in the pool of circulating mycobacterial strains.

The identified ‘’New’’ strain of the lineage 2(EAS) deserves particular attention and will need to be characterized further. Lineage 2 is in general still very rare in Ethiopia (39). The Beijing family has previously been reported to be prone to drug resistance.Strains within this lineage are reported to rapidly adapt to environments with variable stress conditions including antibiotics (31). With increased trade and travel between East Asia and Ethiopia, the risk of multiple introductions of Lineage 2 into the country remains high. It is important to pay special attention to this risk, monitor detected strains and conduct further studies with broader context to better design targeted control efforts.

Strains without assigned spoligotype so-called ‘'New'' made up 29.3% (22/75), and existed in similar spoligotype patterns containing 2 and 6 isolates each. The epidemiological significance of these strains needs further investigation using clinical and molecular tools with better discriminatory power.

Lineage 3(CAS-Delhi, the principal family, or CAS) (with 11 strains in a group) has been reported as the dominant mycobacterial strain circulating in Sudan (32). This indicates cross-border transmission given that people from the current study area share market places with and has free cross-border movement into Sudan (40).

The dominant prevalence of lineage 4 (EA) and lineage 3 (EAI) has already been shown in previous studies from Ethiopia (22, 33). Lineage 1(IO, ancestral) seems to be more prevalent in southern regions of Ethiopia and may be shared with the Eastern African coast and Kenya (41).This again illustrates the need to investigate the molecular epidemiology of M.tuberculosis in the vast border regions of the country to better understand trends and respond to emerging challenges

The observed magnitude of MDR-TB and resistance to anyone first-line anti-TB drug is comparable while Isoniazid monoresistance is considerably higher. Importantly Rifampicin, Streptomycin and Ethambutol monoresistance were lower than in most previous studies elsewhere in the country (34 - 38). The reasons need to be investigated but could include factors related to the study design, time period, sample size or study population (Urban/rural, access to drugs, health seeking behaviour, stigma, mobility of population) among others.

The study has several limitations. Our sample size was relatively small and the yeild of culture was further reduced because of logistic challenges. Nevertheless, the number and diversity of isolates detected provided adequate data for very interesting findings and meaningful conclusions. We relied on spoligotyping alone to detect transmission trends, a method that tends to overestimate rates and indicates older events than whole genome sequencing which is increasingly accepted as a new gold standard. Nevertheles, although the indications from spoligotyping may lack precision in the maginitude of transmission, the interpretation we made from the findings that there is active ongoing transmission in the area is valid (42).

In summary, the findings indicate that the peripheral regions of the country where population density is relatively low in rural settings but urban centres have a high mobility and mix as hubs of trade and travel, where cross border movements are frequent and where health care services are generally scanty and inadequate, there is much diversity but also clustering in circulating *M. tuberculosis* strains. There are several yet unknown strain types whose significance in driving tuberculosis is unexplored. In addition, there are indications that multi drug resistance is emerging and a strain that belongs to Lineage 2 detected. It is therefore important to build local DST facilities in the area to detect and treat MDR TB early and to monitor the molecular epidemiology of tuberculosis in the Benishangul Gumuz and similar regions closely in order to predict trends and respond with appropriate interventions early.**Ethical approval**

The research proposal was evaluated and approved by the health research and ethics review committee of Addis Ababa University, Medical Faculty, Department of Microbiology Immunology and Parasitology; and AHRI/ALERT research review committee with project Reg. No. PO12/13 (S1 File). Institutional support letters were obtained from regional, zonal, and local health administrations. Study participants were provided with sufficient information, and only volunteers were recruited after signing informed consent. DST results were communicated as soon as available to the relevant care giver for better and timely management of patients. MDR-TB cases identified were referred to either the MDR-TB treatment centre at St. Peter TB specialized hospital, Addis Ababa or to Gondar University Hospital MDR TB facility, Gondar.

**Funding source**

This work is fully supported by AHRI core budget. AHRI receives core financial support from Swedish International Development Cooperation Agency (SIDA), Norwegian Agency for Development Cooperation (NORAD), and from the Government of Ethiopia through Ministry of Health. **AA** holds a grant from Human Hereditary and Health in Africa (H3Africa) [H3A/18/003] implemented by the African Academy of Sciences (AAS) and the NEPAD Agency's Alliance for Accelerating Excellence in Science in Africa (AESA) in partnership with Wellcome and a grant from the National Institutes of Health (NIH) Fogarty International Center Global Infectious Diseases entitled “Ethiopia-Emory TB Research Training Program” (D43TW009127).

**Conflict of interests**

Authors declared that there is no conflict of interest.

**Acknowledgements**

The authors would like to thank all the study participants. We would also like extend our gratitude to Mr Shiferaw Bekele for his assistance and guidance during laboratory work, and Mr Legesse Negash during data entry and statistical analysis. We are thankful to Medical laboratory professionals and Nurses working at TB clinics of the study area for their commitment and dedicated assistance during data collection. Lastly, we would like to mention and appreciate that financial and logistic support for the transportation of the identified MDR-TB patients was facilitated by Benishangul Gumuz regional health bureau and Pawe woreda TB program coordinating office.

**Author’s note**

As part of the intervention, the lead author of this study organized community-based health information dissemination program at the area where MDR-TB cases were identified. The participants were TB Program coordinators, administrators of the area, public health institution managers and privately drug vendor owners (S2 File).

**Author Contributions**

**TAL, AA** and **DB** (conceived and designed the experiments) **TAL** (performed all the experiments, analysed the data, and wrote the manuscript) **TAL, YW**, **DA**, **AA** and **DB** (supervision and Project administration, and approval) **MB** (supervision, review & edit manuscript, approval), all authors read and approved the final manuscript.

**References**

1. World Health Organization. Global Tuberculosis Report 2018. Available: <https://www.who.int/tb/publications/global_report/en/>. Accessed 19 March 2019
2. Nahid P, Pai M, Hopewell PC. Advances in the diagnosis and treatment of tuberculosis. Proc Am Thorac Soc. 2006;3(1):103-10.
3. The Federal Ministry of Health Ethiopia. First Ethiopian national population-based Tuberculosis Prevalence Survey.2011. Available: https://www.ephi.gov.et/. Accessed 13 September 2018.
4. World Health Organization. Global tuberculosis report. 2015. Available: <http://apps.who.int/iris/bitstream/10665/191102/1/9789241565059_eng.pdf?ua=1>. Accessed 13 September 2018.
5. World Health Organization. Global tuberculosis report. 2016. Available: <https://apps.who.int/medicinedocs/documents/s23098en/s23098en.pdf> Accessed13 September 2018.
6. Gebreegziabher SB, Bjune GA, Yimer SA. Total Delay Is Associated with unfavourable Treatment Outcome among Pulmonary Tuberculosis Patients in West Gojjam Zone, Northwest Ethiopia: A Prospective Cohort Study. Plos One. 2016; 11(7).
7. Ramage HR, Connolly LE, Cox JS. Comprehensive Functional Analysis of Mycobacterium tuberculosis Toxin-Antitoxin Systems: Implications for Pathogenesis, Stress Responses, and Evolution. PLOS Genetics. 2009; 5(12).
8. Stucki D, Brites D, Jeljeli L, Coscolla M, Liu Q, Trauner A, et al. Mycobacterium tuberculosis lineage 4 comprises globally distributed and geographically restricted sublineages. Nat Genet. 2016;48(12):1535-43.
9. Gagneux S, Small PM. Global phylogeography of Mycobacterium tuberculosis and implications for tuberculosis product development. Lancet Infect Dis. 2007;7(5):328-37.
10. Kamerbeek J, Schouls L, Kolk A, van Agterveld M, van Soolingen D, Kuijper S, et al. Simultaneous detection and strain differentiation of Mycobacterium tuberculosis for diagnosis and epidemiology. J Clin Microbiol. 1997; 35(4):907–14.
11. Shabbeer A, Cowan LS, Ozcaglar C, Rastogi N, Vandenberg SL, Yener B, et al. TB-Lineage: an online tool for classification and analysis of strains of Mycobacterium tuberculosis complex. Infect Genet Evol. 2012;12(4):789-97
12. Couvin D, David A, Zozio T, Rastogi N. Macro-geographical specificities of the prevailing tuberculosis epidemic as seen through SITVIT2, an updated version of the Mycobacterium tuberculosis genotyping database. Infection Genetics and Evolution. 2019; 72:31-43.
13. Central statistical agency (CSA). Population and Housing Census of Ethiopia: Results for Benishangul-Gumuz Region, Vol. 1; 1994. Available: www.csa.gov.et/census-report/complete-report/census-1994.
14. Unicef-Ethiopian Country office. Benishangul-Gumuz Briefing Note. 2016. Available: <https://www.unicef.org/ethiopia/ECO/Benishangul/Gumuz/Brieifing/Note.2016>. Accessed 13.09.2018.
15. World Health Organization. Guidelines for surveillance of drug resistance in tuberculosis. 2009; 4^th^ ed. Available: <https://www.who.int/tb/publications/surveillance_guidelines/en/>. Accessed 13.09.2018
16. Kubica GP, Dye WE, Cohn ML, Middlebrook G. Sputum digestion and decontamination with N-acetyl-L-cysteine-sodium hydroxide for culture of mycobacteria. Am Rev Respir Dis. 1963;87:775-9.
17. Global Laboratory initiative. Mycobacteriology laboratory manual. 2014:47-51.
18. van Klingeren B, Dessens-Kroon M, van der Laan T, Kremer K, van Soolingen D. Drug susceptibility testing of Mycobacterium tuberculosis complex by use of a high-throughput, reproducible, absolute concentration method. J Clin Microbiol. 2007;45(8):2662-8.
19. Wedajo W, Schon T, Bedru A, Kiros T, Hailu E, Mebrahtu T, et al. A 24-well plate assay for simultaneous testing of first and second line drugs against Mycobacterium tuberculosis in a high endemic setting. BMC Res Notes. 2014;7:512.
20. Parsons LM, Brosch R, Cole ST, Somoskovi A, Loder A, Bretzel G, et al. Rapid and simple approach for identification of Mycobacterium tuberculosis complex isolates by PCR-based genomic deletion analysis. J Clin Microbiol. 2002;40(7):2339-45.
21. van Embden JD, van Gorkom T, Kremer K, Jansen R, van Der Zeijst BA, Schouls LM. Genetic variation and evolutionary origin of the direct repeat locus of Mycobacterium tuberculosis complex bacteria. J Bacteriol. 2000;182(9):2393-401.
22. Mihret A, Bekele Y, Aytenew M, Assefa Y, Abebe M, Wassie L, et al. Modern lineages of Mycobacterium tuberculosis in Addis Ababa, Ethiopia: implications for the tuberculosis control programe. African Health Sciences. 2012;12(3):339-44.
23. Esmael A, Wubie M, Desta K, Ali I, Endris M, et al. Genotyping and Drug Resistance Patterns of *M. tuberculosis* in Eastern Amhara region, Ethiopia. J Clin Diagn Res. 2014; 2:102.
24. Garedew L, Mihret A, Mamo G, Abebe T, Firdessa R, Bekele Y, et al. Strain diversity of mycobacteria isolated from pulmonary tuberculosis patients at Debre Birhan Hospital, Ethiopia. Int J Tuberc Lung D. 2013;17(8):1076-81.
25. Diriba B, Berkessa T, Mamo G, Tedla Y, Ameni G. Spoligotyping of multidrug-resistant Mycobacterium tuberculosis isolates in Ethiopia. Int J Tuberc Lung D. 2013;17(2):246-50.
26. Ford CB, Shah RR, Maeda MK, Gagneux S, Murray MB, Cohen T, et al. Mycobacterium tuberculosis mutation rate estimates from different lineages predict substantial differences in the emergence of drug-resistant tuberculosis. Nature Genetics. 2013;45(7):784-90.
27. Alemu J, Mamo G, Ameni G, Pal M. Molecular Epidemiology of Bovine Tuberculosis in Cattle and its Public Health Implications in Gambella Town and its Surroundings, Gambella Regional State, Ethiopia. Molecular Microbiology Research. 2016; 6(1): 1-15.
28. Nuru A, Mamo G, Worku A, Admasu A, Medhin G, Pieper R, et al. Genetic Diversity of Mycobacterium tuberculosis Complex Isolated from Tuberculosis Patients in Bahir Dar City and Its Surroundings, Northwest Ethiopia. Biomed Res Int. 2015;2015:174732.
29. Maru M, Mariam SH, Airgecho T, Gadissa E, Aseffa A. Prevalence of Tuberculosis, Drug Susceptibility Testing, and Genotyping of Mycobacterial Isolates from Pulmonary Tuberculosis Patients in Dessie, Ethiopia. Tuberc Res Treat. 2015;2015:215015.
30. Central statistical agency of Ethiopia. Population and Housing Census of Ethiopia: Results for Benishangul-Gumuz Region, Vol. 1; 1994. Accessed from www.csa.gov.et/census-report/complete-report/census-1994.
31. Ebrahimi-Rad M, Bifani P, Martin C, Kremer K, Samper S, Rauzier J, et al. Mutations in putative mutator genes of Mycobacterium tuberculosis strains of the W-Beijing family. Emerg Infect Dis. 2003;9(7):838-45.
32. Sharaf Eldin GS, Fadl-Elmula I, Ali MS, Ali AB, Salih AL, Mallard K, et al. Tuberculosis in Sudan: a study of Mycobacterium tuberculosis strain genotype and susceptibility to anti-tuberculosis drugs. BMC Infect Dis. 2011;11:219.
33. Agonafir M, Lemma E, Wolde-Meskel D, Goshu S, Santhanam A, Girmachew F, et al. Phenotypic and genotypic analysis of multidrug-resistant tuberculosis in Ethiopia. Int J Tuberc Lung Dis. 2010;14(10):1259-65.
34. Demissie M, Gebeyehu M, Berhane Y. Primary resistance to anti-tuberculosis drugs in Addis Ababa, Ethiopia. Int J Tuberc Lung Dis. 1997;1(1):64-7.
35. Tessema B, Beer J, Emmrich F, Sack U, Rodloff AC. First- and second-line anti-tuberculosis drug resistance in Northwest Ethiopia. Int J Tuberc Lung Dis. 2012;16(6):805-11.
36. Kassu D, Daniel A, Eshetu L, Mekdes G, Beniam F. Drug susceptibility of *Mycobacterium tuberculosis* isolate from smear-negative pulmonary tuberculosis patients, Addis Ababa, Ethiopia. Ethiop J Health Dev. 200822(2):212-215
37. Yimer SA, Agonafir M, Derese Y, Sani Y, Bjune GA, Holm-Hansen C. Primary drug resistance to anti-tuberculosis drugs in major towns of Amhara region, Ethiopia. APMIS. 2012;120(6):503-9.
38. Bruchfeld J, Aderaye G, Palme IB, Bjorvatn B, Ghebremichael S, Hoffner S, et al. Molecular epidemiology and drug resistance of Mycobacterium tuberculosis isolates from Ethiopian pulmonary tuberculosis patients with and without human immunodeficiency virus infection. J Clin Microbiol. 2002;40(5):1636-43.
39. Firdessa R, Berg S, Hailu E, Schelling E, Gumi B, Erenso G, et al. Mycobacterial lineages causing pulmonary and extrapulmonary tuberculosis, Ethiopia. Emerg Infect Dis. 2013;19(3):460-3.
40. Comas I, Hailu E, Kiros T, Bekele S, Mekonnen W, Gumi B, et al. Population Genomics of Mycobacterium tuberculosis in Ethiopia Contradicts the Virgin Soil Hypothesis for Human Tuberculosis in Sub-Saharan Africa. Curr Biol. 2015;25(24):3260-6.
41. Mbugi EV, Katale BZ, Streicher EM, Keyyu JD, Kendall SL, Dockrell HM, et al. Mapping of Mycobacterium tuberculosis Complex Genetic Diversity Profiles in Tanzania and Other African Countries. PLoS One. 2016;11(5):e0154571.

**Supporting information**

**S1 Fig.** Colony characteristic used for identification of the growth of *Mycobacterium tuberculosis*

**S2 Fig**. PCR for RD9 Deletion typing

**S1 File**. Ethical review committee approval sheet

**S2 File.** Regional health bureau certificate

**S1 Table**. Frequency of *M. tuberculosis* family and Lineages
