# Supplementary material for: Genetic diversity and drug resistance pattern of Mycobacterium tuberculosis strains isolated from pulmonary tuberculosis patients in the Benishangul Gumuz region and its surroundings, Northwest Ethiopia
Source: PLoS One. 2020 Apr 8;15(4):e0231320. doi: 10.1371/journal.pone.0231320 (PMC7141659; doi:10.1371/journal.pone.0231320)
Supplement: Attachment — Submitted filename: Response for the comments from reviewers and editor.docx [file pone.0231320.s005.docx]

**Response to the journal requirements (Editor) and Reviewers**

**Journal Requirements (response to the editor):**

When submitting your revision, we need you to address these additional requirements.

1. Please ensure that your manuscript meets PLOS ONE's style requirements, including those for file naming. The PLOS ONE style templates can be found at

http://www.journals.plos.org/plosone/s/file?id=wjVg/PLOSOne_formatting_sample_main_body.pdf and <http://www.journals.plos.org/plosone/s/file?id=ba62/PLOSOne_formatting_sample_title_authors_affiliations.pdf>

- As the requirement and requested by academic editor of the journal the authors adhered to templates provided and found it useful in shaping the manuscript.
- To mention title, current address, corresponding author’s initial, fonts of title and subtitles, references, figure and tables and supplementary information among other points were addressed.

2. Thank you for stating the following in the Acknowledgments Section of your manuscript:

'...we would like to mention and appreciate that financial and logistic support for the transportation of the identified MDR-TB patients was facilitated by Benishangul Gumuz regional health bureau and Pawe woreda TB program coordinating office.'

We note that you have provided funding information that is not currently declared in your Funding Statement. However, funding information should not appear in the Acknowledgments section or other areas of your manuscript. We will only publish funding information present in the Funding Statement section of the online submission form.

Please remove any funding-related text from the manuscript and let us know how you would like to update your Funding Statement. Currently, your Funding Statement reads as follows:

'The funders had no role in study design, data collection and analysis, decision to publish, or preparation of the manuscript.'

Please provide an amended Funding Statement that declares *all* the funding or sources of support received during this specific study (whether external or internal to your organization) as detailed online in our guide for authors at http://journals.plos.org/plosone/s/submit-now

Please state what role the funders took in the study. If any authors received a salary from any of your funders, please state which authors and which funder. If the funders had no role, please state: "The funders had no role in study design, data collection and analysis, decision to publish, or preparation of the manuscript."

c. Please include your amended statements within your cover letter; we will change the online submission form on your behalf.

- Funding related text from the acknowledgement section of the manuscript is removed and re-written as follow on line no. 366-368 :-

‘’… Lastly, we would like to mention and appreciate the Benishangul Gumuz regional health bureau and Pawe woreda TB program coordinating offices for facilitating logistics for the transportation of the identified MDR-TB patients to the treatment initiation centers (TICs). ‘’

**Funding statement, (this will be move to the cover letter as mentioned by the academic editor)**

**Amendment to the funding statement declaration is included in the cover letter and stated as follow.**

- ‘’This work is fully supported by AHRI core budget. AHRI receives core financial support from Swedish International Development Cooperation Agency (SIDA), Norwegian Agency for Development Cooperation (NORAD), and from the Government of Ethiopia through Ministry of Health.
- **‘’AA** holds a grant from Human Hereditary and Health in Africa (H3Africa) [H3A/18/003] implemented by the African Academy of Sciences (AAS) and the NEPAD Agency's Alliance for Accelerating Excellence in Science in Africa (AESA) in partnership with Wellcome and a grant from the National Institutes of Health (NIH) Fogarty International Center Global Infectious Diseases entitled “Ethiopia-Emory TB Research Training Program” (D43TW009127).’’
- Any statements related to funding is removed from the manuscript.

3. Please upload **a new copy of Figure 1** as the detail is not clear. Please follow the link for more information: <http://blogs.PLOS.org/everyone/2011/05/10/how-to-check-your-manuscript-image-quality-in-editorial-manager/>

- Figure 1 is checked for its quality using PACE as recommended and clear and the corrected version is uploaded.

4. Please include a **caption for figure 3.**

- Figure 3 is now renamed as ‘Figure 4 ‘and caption is added as ‘’ Figure 4. Spoligotyping pattern of *M. tuberculosis* isolates from pulmonary TB patients from the Benishangul Gumuz region and its surroundings in North West Ethiopia, 2013/14.’’.

5. Your **ethics statement must appear in the Methods section of your manuscript.** If your ethics statement is written in any section besides the Methods, please move it to the Methods section and delete it from any other section. Please also ensure that your ethics statement is included in your manuscript, as the ethics section of your online submission will not be published alongside your manuscript.

- Ethics statement is REVISED and moved to the **method section** (*line n*o. 198-206).

‘**’Ethical statement**

The research review committee from Addis Ababa University and AHRI/ALERT health research and ethics review committee evaluated and approved the proposal with the project Reg. No. PO12/13 (S1 File). Institutional support letters obtained from regional, zonal, and local health administrations. Study participants provided with sufficient information, and only volunteers recruited after signing informed consent. Results from DST was communicated to the relevant caregiver as soon as available, for better and timely management of patients. MDR-TB cases identified were referred to either the MDR-TB treatment center at St. Peter TB specialized hospital, Addis Ababa, or to Gondar University Hospital MDR-TB facility, Gondar.’’

**Response to the reviewers**

Thank you for the valuable and valid comments that helped us to improve our work.

By appreciating, the effort made and time invested to bring these important points to our attention, the authors addressed all the comments from the journal editor as well as reviewers as curiously as possible. Some points, which are relevant but failed to be addressed by the virtue of limited capacity of the authors, were either strongly recommended to be taken into account in the future work plan or mentioned as limitations.

**Reviewer 1.**

1. Comment to compare our results with data from the following published articles of

Disassa H. *et al*., 2015; Tulu B. *et al*., 2018; and Mekonnen D. *et al*, 2019.

- Our results were compared and further discussed using the recommended papers.

Specifically, Disassa H. *et al*., 2015 is cited as Ref. 30, and its findings are compared and discussed in line with our findings as shown on lines no. 326-331,

- Findings from the systematic review papers by Tulu B. *et al.,* 2018 and Mekonnen D. *et al*., 2019 were also cited as Ref. no 13 and 15, respectively; and their findings were compared, and discussed in the document lines number 92-95 and 324-325.
- In both review papers, the results presented regarding the Benishangul Gumuz region (the current study area) were from a single preliminary study by Disassa H. *et al.,* 2015, and the conclusions and recommendations ask for more research in settings like Benishangul Gumuz region, refer to the conclusion from Tulu B. *et al*, 2018 on page 8 of 10.

2. 129 line is not clear what is AHRI, where is this organization located?

- AHRI is defined as Armauer Hansen Research Institute and located at Addis Ababa, Ethiopia. Additional explanation on the funding sources of the institute is also presented and submitted on a separate letter of the funding statement to the academic editor as follow:-

’’This work is fully supported by AHRI core budget. AHRI receives core financial support from Swedish International Development Cooperation Agency (SIDA), Norwegian Agency for Development Cooperation (NORAD), and from the Government of Ethiopia through Ministry of Health’’

3. 135-136 lines are not the correct interpretation, because decontamination does not occur before, but after mixing within 15 minutes, and centrifugation - not 3000 rpm, but 3000 g should be, and temperature? It must be 4 C.
   - This statement is corrected and presented on line 144-146 as follow:-

‘’…… Briefly, an equal volume of NALC-NaOH and sputum samples was left standing for 15 minutes for digestion and decontamination. Sedimentation was achieved by centrifugation at 3000 rpm for 15 minutes at +4^O^C. ‘’

4. 148 - 149 the term minimum inhibitory concentration of a breakpoint is better replaced by critical concentrations
   - The phrase ‘’minimum inhibitory concentrations’’ is replaced by ‘’critical concentrations’’, and indicated on line 156-158 as follow

‘’…. Four drugs, namely Isoniazid, Rifampicin, Streptomycin, and Ethambutol, were used with critical concentration breakpoints of 0.2μg/ml, 1μg/ml, 2μg/ml, and 5μg/ml, respectively.’’

- It is also corrected on line 161

5. 183 error in numbers 81.3% (75/87)
   - This is now corrected and stated on line 214 as ‘’…… 81.3% (87/107) were culture positive and available for further analysis’’. Calculations for % and number were also checked across the whole manuscript and corrected when find.
6. 188 - 189 woman and man were mixed up
   - The statement mentioned as ‘’There were more female patients than male with 3:1 male to female ratio’’ was wrong and replaced by the statement ‘’…. There were more male patients than females with a 3:1 male to female ratio’’ on line no. 219-220.
7. 338 - 339 to talk about «on-going active transmission of tuberculosis» is not right if spoligotyping is used.
   - For the discussions made based on the finding from spoligotyping, revision has been made and conclusive presentations were corrected, as results from the spoligotyping are valid and suggestive but not conclusive.
   - Line 300-302 ‘’…The predominance of clusters within SIT289 (11 isolates) and SIT53 (nine isolates) could strongly suggest an on-going active transmission of tuberculosis in the area’’, and
   - line 52-53 ‘’….Clustered spoligotype patterns suggest on-going active tuberculosis transmission in the area.’’

**Reviewer 2.**

The authors report the **results of *routine drug susceptibility tests*** to first line anti TB drugs. Although cultures and DSTs and Gold standard tests, the authors did not use any molecular method to assess drug resistant TB. For genetic diversity, authors used spoligotyping, which tend to overestimate links between isolates that is inconclusive for transmission routes of TB.

**Other aspects that could be addressed by authors to strength the paper are:**

1) To perform 15 or 24 loci **MIRU-VNTR genotyping** to strengthen data on MTB diversity.

2) To determine the mutations related to resistance to main anti TB drugs i.e. INH and RIF and to compare these **genotyping** results with those of conventional tests (drug susceptibility testing).

The authors strongly agree the need and importance of 15 or 24 loci MIRU-VNTR for genetic typing, and determining genetic mutation to the main drugs tested in study. Nevertheless, we could not able to address this only because technical, logistic and financial issues during the study period.

Some of the reasons and pertinent justifications of our findings are stated below.

a. There was no MIRU-VNTR in Ethiopia during the study period and still there no this facility available. Obviously, this demand to establish collaboration from abroad that may incur us many things and further delay, if it ends successfully.
b. Though this study at the beginning was designed to address only the genetic diversity of *M. tuberculosis* from the Benishangul Gumuz region (where there was no data available then), we decided to add the drug susceptibility profile study of the isolates using indirect proportion method, which is recommended by WHO as a gold standard method. Unlike most studies focusing on either molecular epidemiology or drug resistance alone, our work addressed both issues that strengthened the findings, especially to the current study setting. Despite its importance to complement the observed DST results, our results on drug resistance lack molecular methods to determine which resistance gene the bacteria carry, and this is explained as a limitation ….[line 345-347]
c. Among limitations mentioned and recommendations made to be addressed in the future work for the current settings, molecular epidemiology using molecular tools with high discriminatory power like MIRU-VNTR and WGS were given emphasis.
d. Furthermore, two systematic reviews by Tulu B. *et al*., 2018 (ref. 13), and Mekonnen D. *et al*, 2019 (Ref. 15) which were recently published and extensively addressing the big picture of genetic diversity of *M. tuberculosis* in the country, were mainly based on spoligotyping. Both the reviews used overlapping studies with only few (7/31) studies included by Mekonnen D. *et al*. addressed additional results from MIRU-VNTR/SNP and both failed to compare results from Benishangul Gumuz region because of limited results presented from a single preliminary study by Disassa H. *et al.,* 2015. The authors of these studies were opted to conclude by recommending the need for further study from the region (the current study area). See conclusion by Tulu B. *et al*., 2018 on page 8 of 10.

We therefore, strongly believe making data avail from spoligotyping with interesting findings and suggestive conclusion is valid (Ref. 10 and 38). Moreover, the drug resistance profile of the isolates will influence the need for further interventions and research in the area especially when and where there is no enough information.

3) To add data regarding **TB prevalence in northwest Ethiopia** and give **more details for** the choice of this setting.

**Prevalence of Tuberculosis in Northwest Ethiopia**

- Except the national TB prevalence survey from 2011 (Ref. 3), there no regional TB prevalence for each region in Ethiopia. However, we managed to extrapolated results from individual studies done the surrounding areas to the current study region like Bahir Dar (Ref. 32), Gondar, Bahir Dar and Debremarkos (Ref. 43) West Gojjam zone (Ref.6), Dessie (Ref.34), in East Amhara (Ref. 26) and major towns in Amhara (Ref. 40) were extensively discussed in the paper.
- Findings from the review papers mentioned above and addressing molecular epidemiology of *M. tuberculosis* at the national level by Tulu B. *et al*., 2018 (ref. 13); and Mekonnen D. *et al*, 2019 (Ref. 15) were discussed.
- Moreover, findings from a study by Disassa H., *et a*l., 2015 (Ref. 30) which was based on Spoligotyping of 33 mycobacterial isolates from the similar setting, were compared and discussed as stated on line no. 326-331.

**Details for choosing this setting**

- More details explaining the demographic and geographic parameters of the study setting were presented under ‘’study setting’’ section line no. 100-118. Briefly, the main reason to choose the current setting, as presented in lines 26-28, is ‘’Because of the supposedly high initial cost and limitation of resources, there is limited information about drug resistance patterns and circulating strains in peripheral and emerging regions of Ethiopia.’’ As mentioned in the study setting section, Benishangul Gumuz is one of the emerging regions in the country with severely limited resources in terms of infrastructure including health facilities. In addition, the recent reviews from the country are lacking information on genetic diversity of *M. tuberculosis* from the area and recommended the need for further studies in the emerging regions like Benishangul Gumuz region (Tulu B. et al., 2018 (ref. 13, conclusion remark on page 8 of 10); and Mekonnen D. *et al*., 2019 (Ref. 15)), and therefore, we believe this study helps to call further studies needed to narrow the wider gaps existing in peripheral settings including Benishangul Gumuz region.
- Moreover, there no single study so far presented drug resistance profile of *M. tuberculosis* complemented with strain diversity in Benishangul Gumuz region, EXCEPT single preliminary study on molecular epidemiology based on spoligotyping of 33 isolates (by Disassa H. *et al*., 2015).

4) To **review methodology section,** as they are **too many details of well-known procedures** (for sputa collection and media preparation)...Also, there was **no brief description for PCR targeting the RD9** and **for spoligotyping method**.

- Details from the method section are now removed, and a brief description for PCR targeting the RD9 and Spoligotying method is added. The authors briefly provided basic details of the method for the purpose of simple understanding and clarification.

**Descriptions for PCR targeting RD9 and Spoligotyping**

- Description for PCR targeting Spoligotyping and RD9 were explained on line 172-184 as:-

‘’ *M. tuberculosis* was differentiated from other species of mycobacteria based on PCR targeting the RD9, as previously described by Parsons et al. (Ref.23). A multiplex PCR was designed to amplify and detect the RD9 using the following primers; Forward RD9_FlankFW: 5’-AACACGGTCACGTTGTCGTG-3’, Reverse RD9_FlankRev: 5’-CAAACCAGCAGCTGTCGTTG-3’, and Internal RD9_InternalR: 5’-TTGCTTCCCCGGTTCGTCTG-3’). The PCR product of 396bp was interpreted as RD9 is present indicating *M. tuberculosis*.

The strains were further typed by spoligotyping based on polymorphism in the direct repeat (DR) locus using a method described previously [Ref.10 and 24]. Briefly, the DR region was amplified using primers, DRa: 5’-GGTTTTGGGTCTGACGAC-3’ and DRb: 5’-CCGAGAGGGGACGGAAAC-3’. After PCR amplification, the PCR products were hybridized to 43 spacer oligonucleotides of the corresponding DR region. The hybridization patterns obtained from the reaction were converted into binary and octal formats. A database with a broad set of strains checked for SIT (Spoligotype International Type), their corresponding numbers, family, and lineages, and assigned to the isolates (Ref. 12).’’

5) The authors have to check the % of the resistance to each drug; I have noticed **many discordances** between **the main text** and **table II (i.e. Rif).**

- To make the presentation clear and simple for the readers, we made new table (Table 2A and B) and figure (Figure 3A and 3B). The % of drug resistance to each drug and combination of drugs is now calculated and presented in elaborated fashion.

6) The paper requires extensive **English editing**.

- Comments on English writing were checked and addressed after having reviewed by peers and experts in the area.
