# Supplementary material for: Genetic diversity and drug resistance pattern of Mycobacterium tuberculosis strains isolated from pulmonary tuberculosis patients in the Benishangul Gumuz region and its surroundings, Northwest Ethiopia
Source: PLoS One. 2020 Apr 8;15(4):e0231320. doi: 10.1371/journal.pone.0231320 (PMC7141659; doi:10.1371/journal.pone.0231320)
Supplement: Attachment — Submitted filename: Response for the comments from editor and reviewers.docx [file pone.0231320.s006.docx]

**Response to the reviewers (a minor revision)**

Thank you again for your valuable and valid comments that helped us to improve our work.

The authors find all the comments to be scientifically pertinent. And, therefore, we addressed all as presented below. Comments are indicated by numbering from 1 to 4 and bolded.

**Reviewer 2.**

1. **In methodology section, the authors have to specify that the primer DRa is biotin labeled.**

**Response:** In the manuscript line no. 180 – 181: the primers used for spoligotyping are presented as:

(….DRa (biotin-labeled):5’-GGTTTTGGGTCTGACGAC-3’and DRb: 5’-CCGAGAGGGGACGGAAAC-3’)

2. **For PCR targeting RD9, the authors have to sepcify that the PCR product of 396bp is related to internal primers.**

**Response:** On line 176-178 stated as:-

‘’A PCR product of 396 bp indicates the presence of RD9 which is unique to *M. tuberculosis* and is absent from all other MTB complex members. Thus, selective amplification of the region targeted by the internal primer demonstrates *M. tuberculosis*.’’

3. **The authors have to reformulate the following sentence:" A database with a broad set of strains checked for SIT (Spoligotype International Type), their corresponding numbers, family, and lineages, and assigned to the isolates"**

**Response:** This statement is reformulated and presented on line no. 183-185 as:

‘’The mycobacterial isolates identified were assigned into Spoligotype International Types (SIT), family, and lineages using databases.’’

4. **Is the manuscript presented in an intelligible fashion and written in standard English? No**

**Response:** To improve the intelligibility and comprehensiveness, senior researchers with ample expertise in TB research extensively revised the manuscript; and the manuscript went through additional proofreading process by a native English speaker and senior scientist. The proofreading is indicated on the acknowledgement part (line no. 374-375).
